# Endodontic Epidemiology

**Published:** 2014-03-08

**Authors:** Arash Shahravan, Ali Akbar Haghdoost

**Affiliations:** a *Kerman Oral and Dental Diseases Research Center, Kerman University of Medical Sciences, Kerman, Iran; *; b * The Research Center for Modeling in Health, Institute for future studies in Health, Kerman University of Medical Sciences, Kerman, Iran*

**Keywords:** Endodontics, Epidemiology

## Abstract

Epidemiology is the study of disease distribution and factors determining or affecting it. Likewise, endodontic epidemiology can be defined as the science of studying the distribution pattern and determinants of pulp and periapical diseases; specially apical periodontitis. Although different study designs have been used in endodontics, researchers must pay more attention to study designs with higher level of evidence such as randomized clinical trials.

## Introduction

Many practitioners who are interested in clinical practice, are treating their patients in private offices or public dental clinics. Suppose that a large number of patients suffer from a particular disease, such as tooth fluorosis. After some time, one may come up with a question like: what is the prevalence of fluorosis among the population of this region? Is the prevalence of this phenomenon higher among the people in this region compared to the people of other provinces or countries? And if so, what factors have resulted in such an increase? 

The answers lie in the realm of epidemiology, which is the science studying the disease distribution and factors determining or affecting it [1]. The science of epidemiology helps us understand the distribution of a phenomenon in a particular region and determines its etiology.

In endodontics, the science of epidemiology has a great role in determining the distribution of pulp and periapical diseases and factors affecting them. The most studied diseases are related to the pulp-dentin complex and among these apical periodontitis (AP) is the inflammation of the periodontal tissues adjacent to the apical foramen which is divided into acute and chronic types. To date, various studies have been conducted to determine its prevalence and causative factors, in different parts of the world. The results have predominantly shown a high prevalence rate for apical periodontitis in different communities [2].

***Descriptive and analytical epidemiology***

Epidemiology means determining the extent and distribution of diseases in different communities, which is referred to as descriptive epidemiology, and also is concerned with the evaluation of determinants or causative factors leading to a high or low prevalence of a disease in different populations, which is referred to as analytical epidemiology. In descriptive epidemiology we seek for answers to questions like “*What *kind of individuals have been affected by the disease?” “*Where* has this disease or health problem occurred?” and “*When* has this problem occurred?” [3].

Descriptive epidemiology is usually the first step in recognizing the status of a disease and amount of exposure to it, and might finally result in formation of a hypothesis in the researcher’s mind [4]. For example, when a large number of oral cancer cases is encountered in a particular population, various hypotheses might form, which explain the possible causes of this problem, including the high rate of chewed tobacco [5].

There are many surveys in the realm of descriptive epidemiology at national or provincial levels that are carried out aiming at determining oral and dental health indexes in different parts of the world. Various national surveys have been carried out in Iran to determine oral health indices, the results of which are used in making strategies for promotion of oral health [6-9]. A survey regarding the prevalence of apical periodontitis which is carried out by Asgary *et al.* in 2010, is one of these [10].

Analytical epidemiology is the scientific evaluation of factors influencing the incidence of diseases and health problems. Here the researcher makes an attempt to find relations by testing hypotheses through statistical analyses and tries to find a relation between an independent variable or exposure and a dependent variable or outcome. For example, in the scenario given at the beginning of this article, the prevalence of fluorosis in one region might be due to a high level of fluoride in the drinking water.

***Observational epidemiology and experimental epidemiology***

In observational epidemiology, the researcher observes the effect of exposure in a particular community and does not interfere in how the exposure affects the outcome. This observation might finally result in describing the findings by the researcher (descriptive epidemiology) or it might result in analysis of the relationship between the exposure and outcome with the use of statistical analyses (analytical epidemiology) [4]. For example, suppose a study is conducted to evaluate the two-year success rate of root canal treatments done by dental practitioners in one city. This study is an observational epidemiology because it only describes the situation. In this context, suppose that based on the results of a similar study in a neighboring city, the same researcher concludes that the success rate in his/her city is lower than that of the neighboring city and states that this might be due to lower rate of using the new techniques and instruments in root canal therapy by dentists in his/her city. Based on this hypothesis the researcher decides to find the relation between using new technologies and the success rate of endodontic treatment between the two cities. This part of the study belongs to observational and analytical epidemiology. It is observational epidemiology because the researcher does not tend to determine the rate of using the independent variable, *i.e*. new endodontic techniques, in the two cities and only observes the relationship.

However, in interventional epidemiology the researcher controls the exposure rate in order to evaluate the relationship between exposure and disease. For example, if two different analgesics are prescribed for two groups of patients who have received root canal therapy in order to determine a suitable medicine to control post treatment pain, and then the severity of pain between the two groups is compared, the researcher determines the patients’ medicine and its dosage and in fact interferes in the exposure variable in the two groups.

Parirokh *et al.* carried out a study in order to evaluate the effect of premedication with ibuprofen or indomethacin on the success of inferior alveolar nerve block for root canal treatment [11]. This study can be considered both observational and experimental. If the study was designed as a clinical trial and the researcher determined the type of the medicine taken in each treatment group (which was the case), the study would be experimental; however, if these two medications were the commonly used analgesics and the researcher only observed the success of anesthesia in a cohort study, without having a role in prescribing the medications in each group, the study would be observational.

***Association and causation***

Association between two variables means that any change in one variable results in change(s) in the other. If an increase in one variable, results in an increase in the second one, the association is positive and if it results in a decrease in the other one, the association is negative. This association might have an absolutely haphazard nature, or due to natural factors these variables might undergo simultaneous changes and the association alone might not mean the presence of a causal relation [12]. For example, it appears that root lengths of teeth on two sides of one jaw are almost similar to their opposite counterparts. This is an association between the lengths of these teeth. However, this relation is not causal because a change in length of one tooth does not result in a change in the length of the other tooth.

The guidelines presented here are introduced by Hill and can be used to make a judgment about the causality of one association:

1. **Temporal relationship** **:** Exposure to the etiologic factor should have occurred before the appearance of symptoms and signs. Therefore, longitudinal studies should be used, instead of cross-sectional ones. In order to evaluate a cause-and-effect relation between the necrotic pulp and higher incidence of failure in root canal therapy, a longitudinal cohort or case-control study should be conducted in which root canal therapy is rendered to teeth with vital and necrotic pulps and success or failure of treatment should be evaluated in these two groups. In fact, diagnosis of the pulp status is made prior to the evaluation of treatment success. Clinical trials are considered very reliable longitudinal studies, which cannot be carried out due to ethical considerations in the example given above.
2. **Strength of the association:** The possibility of the presence of a causal relation increases with an increase in correlation between the exposure and the disease.
3. **Dose-response relationship:** Dose-response relationship means that with an increase in the rate of exposure, there is an increase in the incidence of the disease and if such a relation is encountered, it is highly probable that a cause-and-effect relation exists.
4. **Replication of the finding:** If there is a causal relation, it is expected that similar studies with different subjects will yield similar results.
5. **Biologic plausibility:** Biologic plausibility refers to the conformity of the studied relation to the existing biologic principles.
6. **Consideration of alternative explanations:** In order to make a judgment whether a relation is causal or not, the extent to which other researchers have considered other possibilities in their interpretations and to what extent they have been able to consider their role as negative, should be considered as well.

**Cessation of exposure:** When a factor causes a disease it is expected that if it is withdrawn the risk of the disease would decrease. For example, when the transient and severe cold sensitivity in a tooth is due to a carious lesion resulting in reversible pulpitis, it is expected that complete removal of the lesion and placing a proper restoration will eliminate the symptoms.

**Table 1:** Twenty four articles which were collected by a systematic review. Any variable which was defined as related to AP in articles was assigned (+). Any variable which was not related to AP was assigned (-). If there was not any information about a variable, it had been assigned (?) If in “n” articles the association was defined as (+) and in “k” articles it was defined as (-), the total score was “n-k”

**Author [Ref. No.]**	**Year**	**Root filling**	**Carious lesion**	**Quality of RCT**	**Regular dental visit**	**Smoking**	**Socioeconomic** **status**	**Coronal restoration**	**Sex**	**Age**	**Tooth type**	**Single or two visit**
**Kirkevang ** ***et al.*** ** [**14**]**	2003	+	+	+	+	+	-	?	?	-	?	?
**Estrela ** ***et al.*** ** [**15**]**	2008	?	?	+	?	?	?	+	?	?	?	?
**Genc ** ***et al. *** **[**16**]**	2008	?	?	+	?	?	?	?	+	-	+	?
**Paredes-Vieyra [**17**]**	2013	?	?	?	?	?	?	?	?	?	?	-
**López-López [**18**]**	2012	+	?	?	?	?	?	?	+	+	?	?
**Kamberi ** ***et al.*** ** [**19**]**	2011	?	?	+	?	?	?	?	?	+	?	?
**Chala ** ***et al.*** ** [**20**]**	2011	+	+	+	?	?	?	+	?	?	?	?
**de Chevigny ** ***et al.*** ** [**21**]**	2008	?	?	+	?	?	?	?	?	?	+	?
**Segura-Egea ** ***et al. *** **[**22**]**	2008	?	?	?	?	+	?	?	?	?	?	?
**Frisk F ** ***et al.*** ** [**23**]**	2007	?	?	+	?	?	-	?	?	+	?	?
**Chugal ** ***et al. *** **[**24**]**	2007	?	?	?	?	?	?	-	?	?	?	?
**Chen ** ***et al. *** **[**25**]**	2007	?	?	+	?	?	?	?	?	?	?	?
**Kirkevang ** ***et al.*** ** [**26**]**	2007	+	+	-	?	?	?	+	?	?	+	?
**Ridell ** ***et al. *** **[**27**]**	2006	?	?	+	?	?	?	?	?	?	+	?
**Gesi ** ***et al.*** ** [**28**]**	2006	?	?	?	?	?	?	?	?	?	?	-
**Sathorn ** ***et al.*** ** [**29**]**	2006	?	?	?	?	?	?	?	?	?	?	-
**Kabak ** ***et al. *** **[**30**]**	2005	+	?	+	?	?	?	?	?	?	+	?
**Georgopoulou ** ***et al.*** ** [**31**]**	2005	?	?	?	?	?	?	?	?	?	+	?
**Segura-Egea ** ***et al.*** ** [**32**]**	2004	?	?	+	?	?	?	+	?	?	?	?
**Kirkevang ** ***et al.*** ** [**33**]**	2004	+	?	+	?	?	?	+	?	?	+	?
**Ørstavik ** ***et al.*** ** [**34**]**	2004	?	+	+	?	?	?	?	?	+	+	?
**Farzaneh ** ***et al.*** ** [**35**]**	2004	?	?	+	?	?	?	?	?	+	+	?
**Chugal ** ***et al.*** ** [**36**]**	2003	?	?	+	?	?	?	?	?	?	?	?
**Hommez ** ***et al.*** ** [**37**]**	2002	?	?	+	?	?	?	+*	?	?	?	?
**Total Score**		6	4	15	1	3	-2	4	2	3	9	-3

* *In radiographic* *evaluation*

1. **Consistency with other knowledge:** When a causal relation exists it is expected that the findings should be consistent with other data.
2. **Specificity of the association:** The term refers to the association of a particular exposure with a particular disease. This condition is one of the weakest prerequisites and it may be advisable to remove it from the list because smoking and alcohol, for example, are considered a risk factor for various diseases such as cancer [1].

It should be pointed out that the majority of epidemiologists believe that except for “temporal relationship”, refutation of any other principles does not necessarily mean the absence of a causal relationship [13]. In the terminology, it is advisable to use the term “risk factor” only in cases where the relationship is causal and if it is not, using the term “risk indicator” or “risk determinant” is preferred [4]. For example, sugar consumption is a risk factor for tooth decay but in the social status it is a risk indicator for it [4].

***Risk factors and risk indicators for apical periodontitis*** **:** Endodontic treatment is considered successful provided that it can prevent apical periodontitis (AP). Therefore, studying the prevalence and incidence of AP and its co-factors in various populations, is very important. Recognition of these factors can help to predict the success of endodontic treatment and to inform the patients. The factors best known to induce AP are the microorganisms present in the root canal(s). The pulp should be necrotized to let the microorganisms access the root canal. The most common factor leading to the inflammation of the pulp and its subsequent necrosis is dental caries. Several studies have evaluated the risk factors and risk indicators involved in induction of AP. In order to carry out a systematic review of these studies, a search was done in PubMed, using the keywords “risk” and “apical periodontitis” limited to the titles and article abstracts. A total of 57 articles were retrieved and after reviewing the title and the abstract, the full texts of articles were collected, if necessary. At last 24 articles were found to be related. The majority of studies were cross-sectional. Data collected from these articles have been summarized in Table 1. 

The factor most frequently referred to is the absence of proper root canal therapy, which in 16 articles is cited as being causative to apical periodontitis. One article has refuted the presence of such an association. Lack of a proper root canal therapy leads to AP due to the inability to eliminate microorganisms from the root canal system or to prevent its recontamination. If root canal treatment is carried out meticulously and if all the standard principles are met, this factor can be controlled by dentists and AP is expected to be less prevalent in that community.

**Figure 1:**
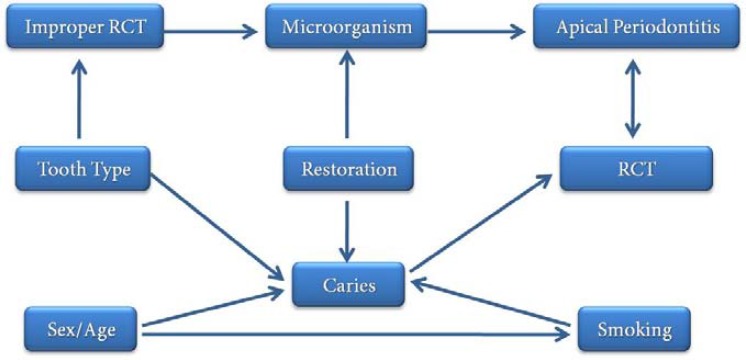
Causal network in the induction of apical periodontitis based on 24 articles listed in Table 1*(MO: micro* *organism*)

**Figure 2:**
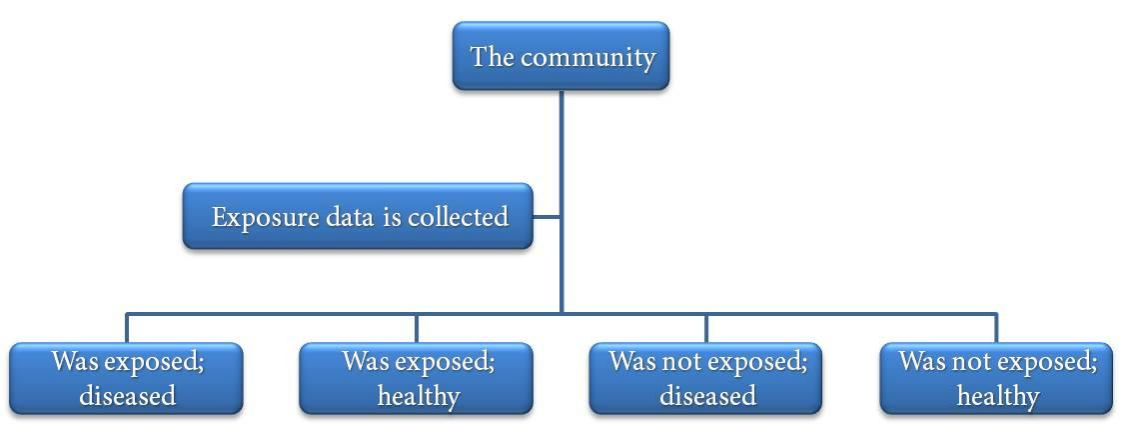
Design of cross-sectional studies

Another factor considered as a risk indicator is the type of the tooth. Nine studies have shown that the prevalence of AP is higher in molars, which seems acceptable because root canal treatment is more difficult and complex in molars; therefore, treatment failure rate is higher in such teeth. In 6 articles, the root canal treatment itself is considered a risk indicator, *i.e*. the odds ratio of AP in endodontically treated teeth is higher than that in teeth without root canal therapy. Since such data was mainly collected from cross-sectional studies, it is not possible to determine whether the teeth underwent root canal therapy and then developed AP or they initially had AP and subsequent to receiving this treatment were still in the healing phase.

Several studies have shown that there are no significant differences between one- and two-visit endodontic treatments in terms of AP incidence. In addition, socioeconomic status has been reported to have no role in AP. The discussion above is shown as a causal network in Figure 1.

***Different types of epidemiologic studies***

Epidemiologic studies can be classified into observational and experimental studies. It is possible to evaluate the effect of one variable on the other, in both categories. As discussed previously, the variable which its effect is being evaluated is referred to as *independent *or* explanatory* variable or exposure; the variable on which the effect of exposure is being evaluated is called the *outcome *or* dependent* variable. The relation between these two variables is referred to as *exposure-outcome association*. 

The most important difference between observational and experimental studies is the control of exposure; not considering, whether the intervention is under control or not. In experimental studies, the exposure is controlled by the researcher. However, in observational studies the researcher’s observations from the exposure-outcome association is described, without interfering with the extent or severity of exposure or deciding on treatment.

***Different types of observational studies***

Observational studies are divided into cross-sectional, case-control and cohort studies.

***Cross-sectional studies: ***In these studies the exposure and outcome are simultaneously evaluated at a certain time in individuals in a community; in fact, they provide a picture of the status of exposure and outcome in individuals in a community. It is obvious that such a study does not have one of the main prerequisites for evaluation of the causal relationship, *i.e*. the temporal relationship, and therefore is the weakest study type for evaluation of exposure-outcome association. For example, if a decision is made to evaluate the relationship between the socioeconomic status and AP in a community, sampling principles are used to select a number of subjects in the community and then their socioeconomic status and presence of AP are simultaneously evaluated. The most important aim of cross-sectional studies is to determine the prevalence of diseases in the community. To date, various cross-sectional studies have been carried out to determine the prevalence of AP in different countries.

**Figure 3:**
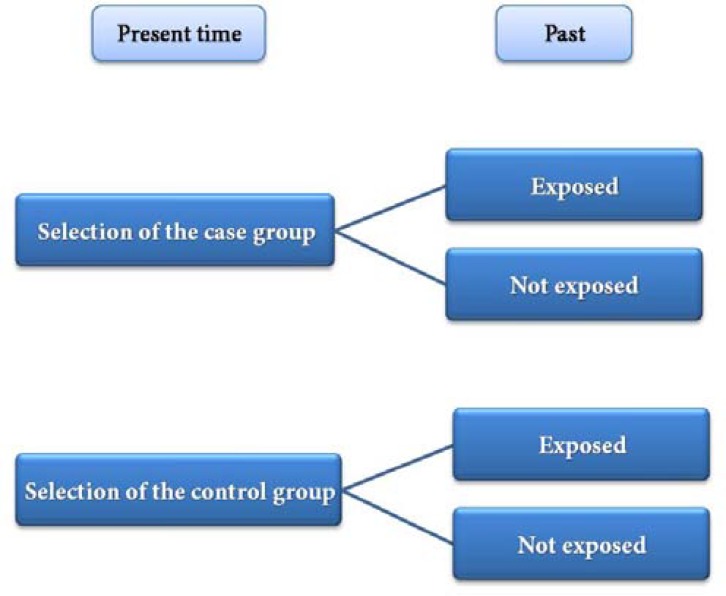
Design of the case-control studies

Pak *et al. *carried out a systematic review of cross-sectional studies on the prevalence of AP in different countries and showed that the prevalence of AP was very high, with at least one radiolucency being detectable in each patient. Also the prevalence of root canal therapy was very high, with almost 2 treatments done for each patient [2]. The general scheme of cross-sectional studies is shown in Figure 2.

In this figure, the data of the 4 boxes at the bottom of the chart can be summarized in a 2×2 table as follows:

**Table:** 

	**Diseased**	**Healthy**
**Exposed**	a	b
**Not exposed**	c	d

The prevalence of the disease in exposed group is *a/a+b *and the prevalence of the disease in individuals without exposure is *c/c+d*. Comparison of these two proportions helps to determine whether the exposure is a risk determinant or not and as discussed previously this kind of comparison does not show a causal relation.

**Figure 4:**
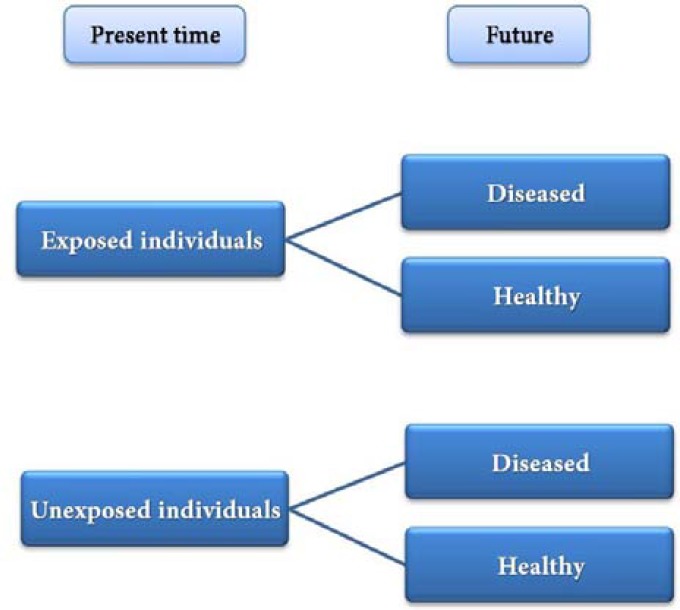
Design of the cohort studies

***Case-control studies: ***Suppose that in a dental clinic, the majority of failed endodontically treated teeth have had necrotic pulps before treatment. Therefore, a hypothesis forms in your mind that a relationship between necrotic pulps and the failure of root canal therapy is likely and can be evaluated. In case-control studies, the history of a group of patients with failed root canal therapy (the case group) is compared with that of patients with successful treatments (the control group) emphasizing on pre-operative necrosis or vitality of the pulp. If the number of teeth with necrotic pulps in failed group (cases) is higher than that in the control group, it might be concluded that pulp necrosis is a risk factor for treatment failure. However, design of such studies has a lot of subtitles, especially in selecting the case and control groups and matching process. These considerations will be explained briefly in the paragraphs to come. Figure 3 shows the steps followed in a case-control study. Considering the chart, it is possible to summarize data in a table, similar to the 2×2 table explained for cross-sectional studies. If the exposure is associated with the disease, it is expected that *a/a+c *will be higher than b/b+d.

**Table:** 

	**Case (diseased)**	**Control (healthy)**	**Total**
**Exposed**	A	B	a+b
**Not exposed**	C	D	c+d
**Total**	a+c	b+d	
**Proportion of exposed subjects**	a/a+c	b/b+d	

Considering the example given above, it is expected that if there is an association between pulp necrosis and failure of treatment, the number of necrotic cases in the case group (failed treatment) will be higher than that in the control group (successful treatment). In case-control studies patients and healthy individuals are selected as the case and control groups, respectively. Then the subjects’ histories are evaluated retrospectively to evaluate the exposure to a specific risk factor in each group. For cases in which there is a very low prevalence rate for a specific disease in a community, case-control studies are carried out better than other research designs. One of the problems which make the accuracy of conclusions questionable in such studies, is the subjects’ ability to remember past exposure to the risk factor, which can result in “recall bias”. For example, if researchers decide to evaluate the relationship between smoking and oral cancer in a case- control study, a group of patients with oral cancer should be selected as a case group and a group of healthy individuals, who are acceptably similar to the patients, should be selected as the controls and both should be questioned about their history of smoking. If the subjects do not provide accurate information about their smoking habits during the previous 5 years or they cannot remember it, a recall bias problem will arise. Note that the information about the subjects’ smoking in the *past* matters not the *present*, because there is a lag period between exposure to tobacco and development of oral cancer.

**Figure 5:**
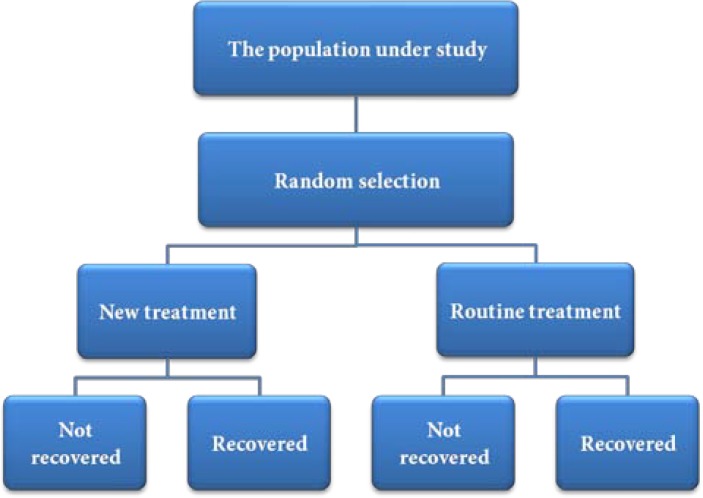
Design of clinical trials

Now suppose researchers decide to evaluate the relationship between apical periodontitis and smoking in a case-control study. López and López carried out such a study, in which patients with AP were assigned to the case group and a number of subjects without AP were assigned to the control group. Then the subjects were questioned about their smoking history. The results revealed a significant relationship between smoking and AP [38].

***Cohort studies: ***Contrary to case-control studies, in cohort design, the study moves from the exposure to the outcome, *i.e*. two groups of subjects with and without exposure are followed to determine the incidence of the disease (outcome). The steps followed in cohort studies are summarized in Figure 4.

Cohort studies have two major types. The first type which is considered as the standard format is known as *prospective cohort study*, in which the researcher selects some subjects with and without exposure at the beginning of the study and follows the two groups for a specific period of time until the symptoms appear or not. The problem with such studies is being time-consuming. For example, a prospective cohort study to evaluate the relation between smoking and oral cancers might take 20 years to yield reliable results, which is practically difficult to carry out. In order to solve such a problem, it has been suggested that a different type of cohort study referred to as *historical/retrospective cohort study,* can be carried out. In this type, two groups of subjects with and without exposure are selected based on their history *e.g.,* smoking, during the past 20 years, and then the disease process, during the past years is

evaluated. In such studies, the study procedure moves from exposure toward outcome but the whole process occurs in the past. However, in the prospective model the study procedure moves from the present to the future. Nevertheless, despite the problems mentioned above, prospective cohort studies are the strongest type of observational studies in establishing a relationship between exposure and outcome.

***Clinical trials: ***There are new topics in epidemiology such as genetic epidemiology, molecular epidemiology, nutritional epidemiology, environmental epidemiology and clinical epidemiology. While the classical definition of epidemiology is the study of the distribution and determinants of diseases in populations, clinical Epidemiology is the use of epidemiology in clinical medicine (treatment, diagnosis). Clinical Epidemiology is the backbone of evidence-based medicine.

The randomized clinical trial is the gold standard of clinical evidence because of minimizing the effects of bias and confounding. Clinical trials are clinical research studies which involve administration of a medication or a therapeutic method in humans. They consist of some types of human studies from those without any control to those sophisticated experimental studies with controls and random allocations. A randomized clinical trial begins with random selection of subjects from a community for treatment with a commonly used technique and a new treatment modality, and then the recovery of patients receiving the new treatment modality is compared with that in patients who have received the routine treatment. The summary of the steps involved is presented in Figure 5.

If the new treatment modality is more efficacious, the outcome is expected to be better in patients receiving it compared to the routinely treated group. For example, various clinical trials have been undertaken to compare the efficacy of various analgesics to relieve pain after root canal therapy.

Designing of clinical trials is very meticulous and involves attention to many details. A summary of these details and considerations which should be observed for meticulous implementation of such studies has been published under the title *Consolidated Standards of Reporting Trials* or “CONSORT guidelines” [39]. Randomization and blindness are the most important considerations in these guidelines.

***Randomization*** **:** Randomization or random allocation means placing subjects in clinical trial groups based on chance, which is allowed in cases with sufficient sample size in each group that allows better matching of the groups before initiation of the study, regarding both known and unknown variables.

***Blindness*** **:** It means that the researcher and/or the participants in the study are not aware of group they belong to. If both the researcher and the participants are blind, the term *double-blind* is used and if one of them is kept unaware, the term “*single-blind*” is indicated. The more the blindness, the less the bias will be. Therefore, some researchers believe that it is even advisable to blind the statistician to the treatment rendered to each group or patient (*triple-blind*). Observing the principles presented in CONSORT guidelines will greatly help researchers in reporting the results of clinical trials.

**Figure 6:**
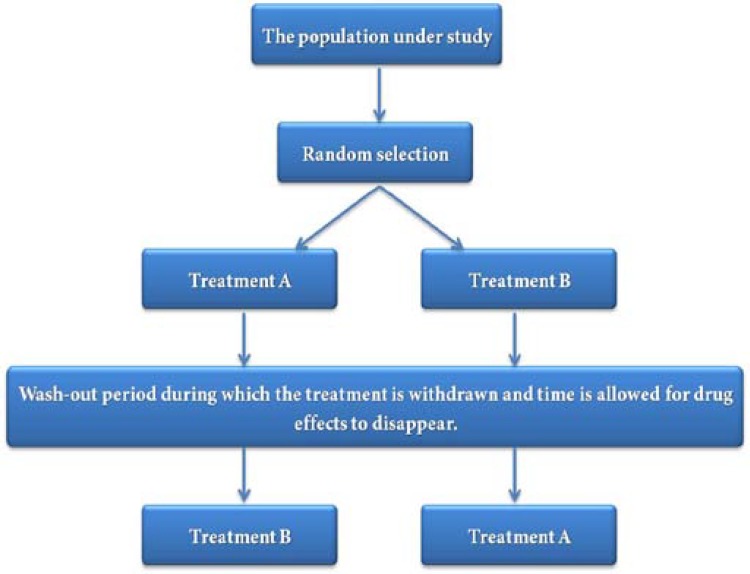
Design of the cross-over studies

Sadeghi evaluated the trend in dental research output in Iran over a period of 20 years (1990-2009) and showed that in terms of studies with a high level of evidence (LoE), only one systematic review was published by Iranian researchers. Of the total number of dental clinical trial articles (11,947) published in PubMed during this period, 74 (0.62%) were written by Iranian authors [40].

One of the most important considerations in performing clinical trials, also discussed in CONSORT guideline, is observing the ethical principles in different stages of these studies. In a study by Navabi *et al.*, the reporting of ethical principles was evaluated in clinical trials published in Iranian dental journals from 2001 to 2011. The results showed that the majority of these articles have not reported important ethical principles. In this context, only 15.3% of these articles have reported obtaining ethical approval for the study [41]. 

In another study, the quality of reporting the sample size calculation in published clinical trials in *Journal of Endodontics* and *International Endodontic Journal* between years 2000-1 and 2009-10 was evaluated. There was a significant increase in reporting the sample size and its calculation and also clinical importance level in 2009-10 compared to 2000-1 [42]. 

The design depicted in Figure 5 is of the parallel type; however, a different type of clinical trial, referred to as cross-over, is common, especially in dentistry. In this type of study, the selected patients are randomly placed in two groups and subjected to different drug regimens. After a period, the treatments rendered to both groups are replaced with each other. This design helps each patient serve as his/her own control and therefore the variables which can affect comparison of the two medications can be managed. The design of a cross-over study is depicted in Figure 6.

**Figure 7:**
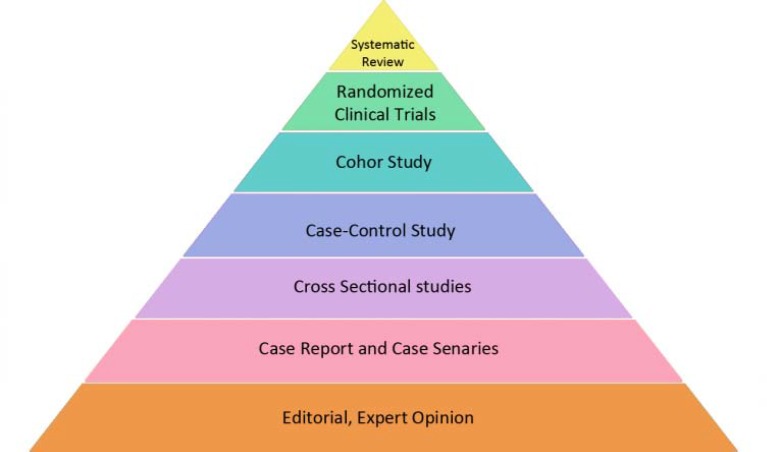
Level of evidence (LoE) pyramid, systematic reviews and randomized clinical trials have the highest level of evidence and editorial and expert opinion have the least

***Other types of studies***

In the following section, some other important types of study designs are discussed which have become more common in recent years.

***Ecological studies***

As a kind of epidemiologic studies, the study unit in *ecological studies* is a group of individuals or a community, rather than a single individual. For example, for evaluating the relation between the average income of a community and the prevalence of AP, this type of study is used; the average income reported by previous studies is used and the individual income of subjects is not questioned. Note that the results of such studies cannot be extended to the individuals in that community (*ecological fallacy*). Such studies are very useful for general decision-making process at provincial or national levels.

***Secondary studies***

Secondary studies are carried out on other studies, so the subjects are the studies carried out by other researchers. One of the most important types of this category are systematic reviews, in which scientific search with precise predetermined criteria is used to collect all high-quality studies about the research question. Then data is retrieved from these studies and, if possible, they are integrated by meta-analysis. If sufficient number of reliable articles are available on the subject, systematic reviews are able to provide the highest level of evidence in relation to the research question.

A total of 151 systematic reviews have been published up to September, 1^st^, 2013 in *Journal of Endodontics, International Endodontic Journal, Dental Traumatology, *and* Oral Surgery, Oral Medicine, Oral Pathology, Oral Radiology*.

A study on systematic reviews, evaluating the quality of meta-analyses carried out in the field of endodontics in 2010, reported the articles considering “A Measurement Tool to Assess Systematic Reviews”, to be of high quality. The most important drawback of assessed systematic reviews was lack of report on publication bias [44].

**Figure 8:**
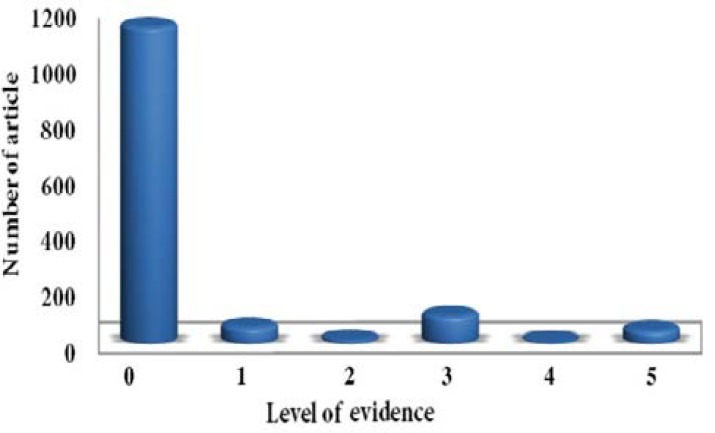
Retrieved from the study by Shafiei and Shahravan [43], the LoE of papers in two leading endodontic journals

***Qualitative research studies: ***All the study types discussed above are *quantitative* and deal with numbers and statistical analyses. However, *qualitative *studies deal with non-numerical data and their aim is to evaluate individual and group characteristics and therefore they are carried out using non-statistical methods. Qualitative research studies are particularly useful and carried out in the field of social epidemiology and evaluation of behaviors [4]. The analysis of data in such studies is different from that in qualitative studies and includes methods such as triangulation, grounded theory analysis, content analysis and narrative data analysis. In a study in 2011 the quality of life after endodontic treatment and implant treatment was compared between two groups of patients. Group discussion technique was used for data collection [45]. In such designs, qualitative technique in the form of in-depth interviews might be better than quantitative techniques such as use of questionnaires. In the content analysis carried out in that study, important issues were evaluated in both treatment modalities, including the importance of general health, financial matters, acceptance of treatment, the time duration necessary to complete treatment and the number of necessary follow-up sessions.

**Level of evidence (LoE) in different studies**

Each type of study discussed above has a level of value to be used in clinical decision-making. In evidence-based medicine a pyramid is depicted, in which different types of studies are classified based on their level of evidence (LoE). As seen in Figure 7, the highest LoE belongs to systematic reviews, followed, in descending order, by randomized clinical trials, cohort studies, case-control studies, cross-sectional studies, case reports and case caries; editorials and expert opinions are located at the lowest end of the spectrum. *In vitro* and animal studies have no place in the evidence pyramid despite their importance, especially in the dental field and testing of new materials, which means that it is not possible to draw clinical conclusions based on their results.

In one study, the LoE in two leading endodontic journals, *i.e.* *Journal of Endodontics* and *International Endodontic Journal*, was evaluated by assessing all the articles published in these two journals in 2000, 2006 and 2010. The results showed that 83.6% of the articles were non-evidence-based and only 3.2% of the articles were clinical trials. However, in recent years, an increase is observed in the number of articles with higher level of evidence in these two journals [43]. Figure 8 shows the number of articles with various levels of evidence published in the aforementioned journals.

Two studies aiming to evaluate the level of evidence in endodontic articles about the outcomes of endodontic retreatment [46] and endodontic surgeries [47], showed that there was only a limited number of studies with a high LoE on these two subjects.

**Bias, confounder and interaction**

Bias, confounder and interaction are factors which lead to errors in interpretation of the results of studies. Therefore, researchers should pay attention to the possible presence of such factors during designing, carrying out and interpretation of results and take them into account.

***Bias: ***This term refers to any systematic error in designing, carrying out or analyzing the results, leading to an error in relation to estimation of the effect of an exposure on an outcome. The usual term is *error*, which means a random error rather than a systematic one. Bias has various types, the most important being *selection *and *information bias.*

***Selection bias: ***This means to select the subjects so that an error in the results of the study occurs. For example, when a researcher decides to estimate the prevalence of TMJ disorders (TMD) in patients living in Kerman, it is not sensible to select subjects from dental clinics, because it results in selection bias. In fact, this type of subject selection results in overestimation of the actual results, as most of the patients referring to dental clinics probably have a problem in their dental region and TMD is not an exception. Therefore, the percentage of individuals with TMD will erroneously be reported greater than the real percentage.

***Information bias: ***This error occurs when the collected data on exposure or outcome or both are not correct. Several factors lead to such an error, including error in remembering, error in interviewing subjects and error in the use of subjects’ medical history. This error has a greater effect when the measurement accuracy is different between the groups under study. For example, if the researcher carrying out measurements is not blind, (none)conscious differences in the accuracy of measuring pain severity as an outcome variable in different treatment groups is expected, which is referred to as *information bias*. Unfortunately, different studies carried out to evaluate the possibility of bias in published dental articles have shown a high percentage of bias. A study on 40 open-access dental journals indexed at Lilacs database showed that out of 4503 clinical trials published from 2002 to 2007, only 10 could be called real clinical trials and only one of them had low bias risk [48].

**Figure 9:**
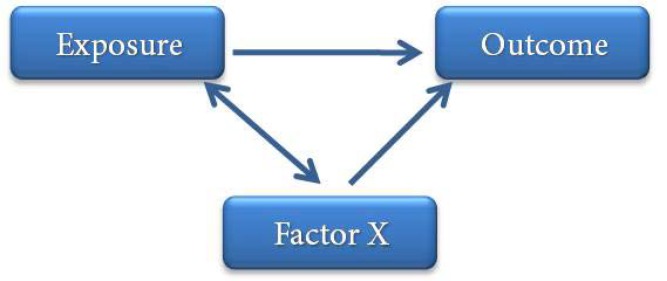
Situation of *Factor X* as the confounding variable

Another study evaluated *publication bias* in dental journals and showed that there is significant interest in these journals to publish articles with results showing statistically significant differences. In this context, 75-90% of the results of articles published in these journals have results with significant differences. This finding was independent of the impact factor of the journals. Publication bias is generally being taken into account in systematic reviews, indicating a more interest to publish articles with significant results [49].

As a key point in designing studies, possibility of non-response bias must be considered. For example a study showed that dentists who do not participate in questionnaire-based designs (non-responders) have information on the subject, different from that of dentists who respond to questionnaires (responders), indicating non-response bias [50].

Literature review did not bring up any articles, specifically evaluating bias in endodontic field and it appears that a research in this respect might be useful.

***Confounding:*** This term refers to a problem in determining the relationship between the exposure and outcome due to the correlation of the exposure with another variable which affects the outcome. In fact, in such situations, a part or all the relation observed between the exposure and outcome is due to another variable which is related to outcome but is not under the influence of the exposure. In other words, the variable X is considered a confounding factor for the relation between exposure and outcome when: *i)* the variable X is a risk factor for the outcome, or *ii)* the variable X has a correlation with exposure but does not result from the exposure. In summary, the most common situation of a confounding variable is shown in Figure 9**. **

Following the same example, we can ask: “Does drinking coffee lead to pancreatic cancer?”. In other words, “Does the risk of pancreatic cancer increase with an increase in drinking coffee?”

**Figure 10:**
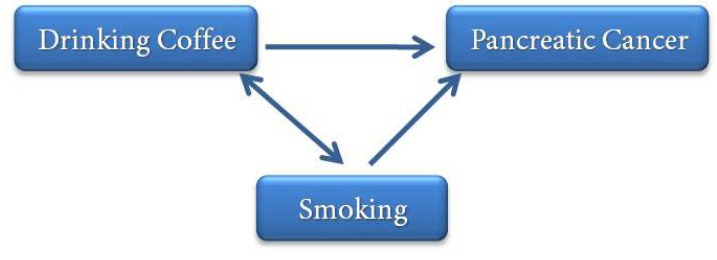
Relation between drinking coffee, smoking and cancer

Evaluations carried out by researchers showed that such a relation exists but another factor is also important in this relationship, which is smoking because is a known risk factor for pancreatic cancer and is correlated with drinking coffee but the relationship is not causal, *i.e*. individuals do not smoke due to drinking coffee; therefore, the relationship can be shown as in Figure 10**. **.

Given the diagram shown above, smoking is a confounding variable for the relation between drinking coffee and pancreatic cancer. Confounding factors can be controlled during designing the study and during analysis of the results. During the designing stage, they can be controlled through restriction, matching, stratification and randomization. During the analysis stage, such factors can be controlled through post-stratification and multi-variable modeling.

 Consider the case of clinical trials in which two different canal preparation techniques are compared with each other in relation to their success percentages. If the severity of AP in the two groups is different at baseline, naturally there is a possibility that even assuming that the two techniques yield similar treatment results; the success percentages will be different between the two groups. In such a situation, the severity of AP at baseline is a confounding factor. The best technique used to solve such a problem is randomization at baseline, which is the only technique of matching the groups in relation to both known and unknown variables. 

**Effect modification**

Effect modification refers to a situation in which the effect of the exposure on the outcome is different at different levels of another variable. For example, age is an effect modifier in various situations because the effect of an exposure on the induction of a disease might be different (low or high) at different ages.

Evaluation of confounding factors and effect modification is not only carried out conceptually and through drawing a causal network but it is also carried out through statistical analyses. These analyses are usually carried out through stratified analysis of effect measure at the level of the third variable suspected of being a confounding factor or an effect modifier. It is always advisable to evaluate effect modification at baseline. If such an effect exists, there is no need for evaluation of confounding effect. One of the Chi-squared group tests is used to evaluate effect modification and differences between the evaluated groups in relation to effect measure. The presence of differences indicates heterogeneity of the groups and the presence of effect modification. In order to evaluate confounding variables, at first the effect measure is calculated at different levels of the third variable and then their weighted mean is obtained using Mantel-Haenszel method. If this cumulative value is different from the primary value of the effect measure, confounding effect exists.

## Conclusion

Endodontic epidemiology has a great role in determining the distribution of pulp and periapical diseases and the factors influencing them. To clarify a causal network in association between risk factors and pulp and periapical diseases, endodontic researchers need to know different study designs in observational and experimental epidemiology. Despite great effort and leaps in endodontics, researches with high level of evidence in endodontics are still few and far between.

## References

[1] Gordis L (2009). Epidemiology.

[2] Pak JG, Fayazi S, White SN (2012). Prevalence of periapical radiolucency and root canal treatment: a systematic review of cross-sectional studies. J Endod.

[3] Mausner JS, Kramer S, Bahn AK (W B Saunders Company). Epidemiology: An Introductory Text.

[4] Chattopadhyay A (2009). Oral Health Epidemiology: Principles and Practice.

[5] Wiener RC (2013). Association of smokeless tobacco use and smoking in adolescents in the United States: An analysis of data from the Youth Risk Behavior Surveillance System survey. J Am Dent Assoc.

[6] Bayat-Movahed S, Samadzadeh H, Ziyarati L, Memary N, Khosravi R, Sadr-Eshkevari PS (2011). Oral health of Iranian children in 2004: a national pathfinder survey of dental caries and treatment needs. EMHJ.

[7] Hessari H, Vehkalahti MM, Eghbal MJ, Samadzadeh H, Murtomaa HT (2008). Oral health and treatment needs among 18-year-old Iranians. Medical Principles and Practice.

[8] Hessari H, Vehkalahti MM, Eghbal MJ, Murtomaa H (2008). Tooth loss and prosthodontic rehabilitation among 35-to 44-year-old Iranians. Journal of oral rehabilitation.

[9] Hessari H, Vehkalahti MM, Eghbal MJ, Murtomaa HT (2007). Oral health among 35-to 44-year-old Iranians. Medical Principles and Practice.

[10] Asgary S, Shadman B, Ghalamkarpour Z, Shahravan A, Ghoddusi J, Bagherpour A, Akbarzadeh Baghban A, Hashemipour M, Ghasemian Pour M (2010). Periapical Status and Quality of Root canal Fillings and Coronal Restorations in Iranian Population. Iran Endod J.

[11] Parirokh M, Ashouri R, Rekabi AR, Nakhaee N, Pardakhti A, Askarifard S, Abbott PV (2010). The effect of premedication with ibuprofen and indomethacin on the success of inferior alveolar nerve block for teeth with irreversible pulpitis. J Endod.

[12] Nasseri K (2010). A comprehensive dictionary of epidemiology.

[13] Szklo M, Nieto J (2007). Epidemiology: Beyond the Basics.

[14] Kirkevang LL, Wenzel A (2003). Risk indicators for apical periodontitis. Community Dent Oral Epidemiol.

[15] Estrela C, Leles CR, Hollanda AC, Moura MS, Pecora JD (2008). Prevalence and risk factors of apical periodontitis in endodontically treated teeth in a selected population of Brazilian adults. Braz Dent J.

[16] Genc Y, Gulsahi K, Gulsahi A, Yavuz Y, Cetinyurek A, Ungor M, Col M (2008). Assessment of possible risk indicators for apical periodontitis in root-filled teeth in an adult Turkish population. Oral Surg Oral Med Oral Pathol Oral Radiol Endod.

[17] Paredes-Vieyra J, Enriquez FJ (2012). Success rate of single- versus two-visit root canal treatment of teeth with apical periodontitis: a randomized controlled trial. J Endod.

[18] Lopez-Lopez J, Jane-Salas E, Estrugo-Devesa A, Castellanos-Cosano L, Martin-Gonzalez J, Velasco-Ortega E, Segura-Egea JJ (2012). Frequency and distribution of root-filled teeth and apical periodontitis in an adult population of Barcelona, Spain. Int Dent J.

[19] Kamberi B, Hoxha V, Stavileci M, Dragusha E, Kuci A, Kqiku L (2011). Prevalence of apical periodontitis and endodontic treatment in a Kosovar adult population. BMC Oral Health.

[20] Chala S, Abouqal R, Abdallaoui F (2011). Prevalence of apical periodontitis and factors associated with the periradicular status. Acta Odontol Scand.

[21] de Chevigny C, Dao TT, Basrani BR, Marquis V, Farzaneh M, Abitbol S, Friedman S (2008). Treatment outcome in endodontics: the Toronto study--phase 4: initial treatment. J Endod.

[22] Segura-Egea JJ, Jimenez-Pinzon A, Rios-Santos JV, Velasco-Ortega E, Cisneros-Cabello R, Poyato-Ferrera MM (2008). High prevalence of apical periodontitis amongst smokers in a sample of Spanish adults. Int Endod J.

[23] Frisk F Epidemiological aspects on apical periodontitis. Studies based on the Prospective Population Study of Women in Goteborg and the Population Study on Oral Health in Jonkoping, Sweden. Swed Dent J Suppl.

[24] Chugal NM, Clive JM, Spangberg LS (2007). Endodontic treatment outcome: effect of the permanent restoration. Oral Surg Oral Med Oral Pathol Oral Radiol Endod.

[25] Chen CY, Hasselgren G, Serman N, Elkind MS, Desvarieux M, Engebretson SP (2007). Prevalence and quality of endodontic treatment in the Northern Manhattan elderly. J Endod.

[26] Kirkevang LL, Vaeth M, Horsted-Bindslev P, Bahrami G, Wenzel A (2007). Risk factors for developing apical periodontitis in a general population. Int Endod J.

[27] Ridell K, Petersson A, Matsson L, Mejare I (2006). Periapical status and technical quality of root-filled teeth in Swedish adolescents and young adults. A retrospective study. Acta Odontol Scand.

[28] Gesi A, Hakeberg M, Warfvinge J, Bergenholtz G (2006). Incidence of periapical lesions and clinical symptoms after pulpectomy--a clinical and radiographic evaluation of 1- versus 2-session treatment. Oral Surg Oral Med Oral Pathol Oral Radiol Endod.

[29] Sathorn C, Parashos P, Messer HH (2005). Effectiveness of single- versus multiple-visit endodontic treatment of teeth with apical periodontitis: a systematic review and meta-analysis. Int Endod J.

[30] Kabak Y, Abbott PV (2005). Prevalence of apical periodontitis and the quality of endodontic treatment in an adult Belarusian population. Int Endod J.

[31] Georgopoulou MK, Spanaki-Voreadi AP, Pantazis N, Kontakiotis EG (2005). Frequency and distribution of root filled teeth and apical periodontitis in a Greek population. Int Endod J.

[32] Segura-Egea JJ, Jimenez-Pinzon A, Poyato-Ferrera M, Velasco-Ortega E, Rios-Santos JV (2004). Periapical status and quality of root fillings and coronal restorations in an adult Spanish population. Int Endod J.

[33] Kirkevang LL, Vaeth M, Wenzel A (2004). Tooth-specific risk indicators for apical periodontitis. Oral Surg Oral Med Oral Pathol Oral Radiol Endod.

[34] Orstavik D, Qvist V, Stoltze K (2004). A multivariate analysis of the outcome of endodontic treatment. Eur J Oral Sci.

[35] Farzaneh M, Abitbol S, Lawrence HP, Friedman S (2004). Treatment outcome in endodontics-the Toronto Study. Phase II: initial treatment. J Endod.

[36] Chugal NM, Clive JM, Spangberg LS (2003). Endodontic infection: some biologic and treatment factors associated with outcome. Oral Surg Oral Med Oral Pathol Oral Radiol Endod.

[37] Hommez GM, Coppens CR, De Moor RJ (2002). Periapical health related to the quality of coronal restorations and root fillings. Int Endod J.

[38] Lopez-Lopez J, Jane-Salas E, Martin-Gonzalez J, Castellanos-Cosano L, Llamas-Carreras JM, Velasco-Ortega E, Segura-Egea JJ (2012). Tobacco smoking and radiographic periapical status: a retrospective case-control study. J Endod.

[39] Schulz KF, Altman DG, Moher D (2010). CONSORT 2010 Statement: Updated guidelines for reporting parallel group randomised trials. J Clin Epidemiol.

[40] Sadeghi M, Shahravan A, Haghdoost AA, Asgary S, Rad M (2012). Trend in dental research output in Iran over a period of 20 years (1990-2009). Int Dent J.

[41] Navabi N, Shahravan A, Modaberi A (2013). Reporting of Ethical Considerations Associated with Clinical Trials Published in Iranian Dental Journals between 2001 and 2011. Iran J Public Health.

[42] Shahravan A, Rad M, Hashemipour M, Sharifi M, Haghdoost A (2014). Sample size calculation in published clinical trials of twoleading Endodontic journals. Iran Endod J.

[43] Shafiei L, Shahravan A (2013). The level of evidence in two leading endodontic journals. Iran Endod J.

[44] Suebnukarn S, Ngamboonsirisingh S, Rattanabanlang A (2010). A systematic evaluation of the quality of meta-analyses in endodontics. J Endod.

[45] Gatten DL, Riedy CA, Hong SK, Johnson JD, Cohenca N (2011). Quality of life of endodontically treated versus implant treated patients: a University-based qualitative research study. J Endod.

[46] Paik S, Sechrist C, Torabinejad M (2004). Levels of evidence for the outcome of endodontic retreatment. J Endod.

[47] Mead C, Javidan-Nejad S, Mego ME, Nash B, Torabinejad M (2005). Levels of evidence for the outcome of endodontic surgery. J Endod.

[48] Ferreira CA, Loureiro CA, Saconato H, Atallah AN (2011). Assessing the risk of bias in randomized controlled trials in the field of dentistry indexed in the Lilacs (Literatura Latino-Americana e do Caribe em Ciencias da Saude) database. Sao Paulo Med J.

[49] Polychronopoulou A, Pandis N, Eliades T (2010). Assessment of publication bias in dental specialty journals. J Evid Based Dent Pract.

[50] Shelley AM, Brunton P, Horner K (2012). Questionnaire surveys of dentists on radiology. Dentomaxillofac Radiol.

